# Differences in Distribution and Detection Rate of the [^68^Ga]Ga-PSMA Ligands PSMA-617, -I&T and -11—Inter-Individual Comparison in Patients with Biochemical Relapse of Prostate Cancer

**DOI:** 10.3390/ph15010009

**Published:** 2021-12-22

**Authors:** Falk Gühne, Stefanie Radke, Thomas Winkens, Christian Kühnel, Julia Greiser, Philipp Seifert, Robert Drescher, Martin Freesmeyer

**Affiliations:** Clinic of Nuclear Medicine, Jena University Hospital, Am Klinikum 1, 07747 Jena, Germany; falk.guehne@med.uni-jena.de (F.G.); stefanie.radke@med.uni-jena.de (S.R.); thomas.winkens@med.uni-jena.de (T.W.); christian.kuehnel@med.uni-jena.de (C.K.); julia.greiser@med.uni-jena.de (J.G.); philipp.seifert@med.uni-jena.de (P.S.); robert.drescher@med.uni-jena.de (R.D.)

**Keywords:** PSMA, PET/CT, prostate cancer, biochemical relapse, ^68^Gallium

## Abstract

The biochemical relapse of prostate cancer is diagnostically challenging but of high clinical impact for subsequent patient treatment. PET/CT with radiolabeled PSMA ligands outperforms conventional diagnostic methods in the detection of tumor recurrence. Several radiopharmaceuticals were and are available for use. The aim of this study was to investigate whether the routinely applied [^68^Ga]Ga-PSMA ligands PSMA-617, -I&T and -11 (HBED-CC) differ in physiological and pathological distribution, or in tumor detection rate. A retrospective evaluation of 190 patients (39 patients received PSMA-617, 68 patients PSMA-I&T and 83 patients PSMA-11) showed significant differences in tracer accumulation within all organs examined. The low retention within the compartments blood pool, bone and muscle tissue is a theoretical advantage of PSMA-11. Evaluation of tumor lesion uptake and detection rate did not reveal superiority of one of the three radiopharmaceuticals, neither in the whole population, nor in particularly challenging subgroups like patients with very low PSA levels. We conclude that all three [^68^Ga]Ga-PSMA ligands are equally feasible in this clinically important scenario, and may replace each other in case of unavailability or production restrictions.

## 1. Introduction

Tumor recurrence of prostate cancer is common and challenging for diagnostics and treatment [1]. Regarding patients for whom a curative therapeutic approach is chosen, surgical prostatectomy and definitive radiotherapy are available treatment options [2]. Depending on the initial treatment, a tumor relapse can occur in 20–50% [3,4,5]. Initial tumor staging and differentiation (i.e., Gleason score) are well-known prognostic indicators [2]. The serum level of prostate-specific antigen (PSA) is an important marker in the follow-up care of prostate cancer [6]. An increase in the PSA level after temporary suppression—a so-called biochemical relapse—indicates a recurrence of the tumor disease. The detection of the structural correlate, and therefore the differentiation between local and systemic tumor recurrence, is decisive for the subsequent treatment of the patient [7,8]. Multiple diagnostic modalities like ultrasound, CT, MRI, bone scan or biopsy are available to reveal tumor manifestations, but frequently remain insufficient for this purpose [9,10].

Positron emission tomography/computed tomography (PET/CT) addressing prostate-specific membrane antigen (PSMA) has been established in the last decade and is gaining relevance in the diagnostics and therapy of prostate cancer [11,12,13,14]. The target structure PSMA is a cell membrane protein, which is markedly—albeit not specifically—overexpressed on prostate cancer cells [15,16,17]. Despite its increasing importance, especially in the pre-therapeutic evaluation of patients with metastatic carcinoma, the recommendation in guidelines and the widespread availability of PSMA-PET/CT are still limited [6,18,19,20]. Recently, several different ^68^Ga- and ^18^F-radiolabeled PSMA ligands have been introduced depending on in-house production conditions, commercial availability and combinability with therapeutic markings in the context of PSMA-radioligand therapy (RLT). Especially ^68^Ga-labeled radiopharmaceuticals are comprehensively used for PET/CT diagnostics. In our tertiary-care PET/CT center, the three ligands PSMA-617 (*psma*-2-naphthyl-L-Ala-cyclohexane-DOTA), PSMA-I&T (*psma*-[(Sub)DLys-DPhe-DTyr(3I)-DOTAGA]) and PSMA-11 (*psma*-(Ahx)-HBED-CC) have been applied at different time intervals over the past few years. PSMA-11 has become the current clinical standard of ^68^Ga-labeled tracers and has been approved by the US FDA lately [21]. Comparisons have been made of the distribution and diagnostic performance of the radiopharmaceuticals, especially with regard to the newly established [^18^F]F-PSMA ligands and the formerly used choline-addressing tracers [22,23,24,25,26]. This is the first clinical comparison of these three [^68^Ga]Ga-PSMA ligands with each other.

The aim of this study was to compare the biodistribution of the ^68^Ga-labeled PSMA ligands 617, I&T and 11 in non-tumor compartments and in tumor lesions, and to evaluate the influence of the biodistribution on the detection rate of tumor lesions in patients suffering from the biochemical recurrence of prostate cancer. A further aim of the study was to determine whether one of the tracers is preferable for this special clinical issue.

## 2. Results

### 2.1. Patients

According to the methodological criteria, 190 men were included in the study. The patients had a mean age of 69.2 ± 6.7 years, an initial median Gleason score of 7 (interquartile range of 1) and a present PSA level of 4.5 ± 11.2 ng/mL. Sixty-one (32%) patients had a PSA level ≤0.5 ng/mL, 20 (11%) >0.5–1 ng/mL, 33 (17%) > 1–2 ng/mL and 76 (40%) >2 ng/mL. The initial definitive tumor treatment had been radical prostatectomy in 84% and radiotherapy in 16% of cases, respectively. Thirty-nine percent of patients had an ongoing or prior ADT. Thirty-nine patients received [^68^Ga]Ga-PSMA-617, 68 patients [^68^Ga]Ga-PSMA-I&T and 83 patients [^68^Ga]Ga-PSMA-11. Patients’ characteristics are shown in Table 1. None of the clinical features yielded an unequal distribution of variables between the tracer groups. Whereas the overall model for ADT was without significant difference, ADT had been applied in patients receiving PSMA-617 more often than in those receiving PSMA-11 (*p* = 0.024) and PSMA-I&T (*p* = 0.032), respectively.

PET/CT parameters were according to intra-clinical standards: 241.9 ± 32.5 MBq were administered, without differences between the PSMA-617, PSMA-I&T and PSMA-11 tracer groups with 250.4 ± 11.6, 235.4 ± 35.4 and 243.2 ± 35.8 MBq, respectively (*p* = 0.059). The time to the start of the PET acquisition was unintentionally significantly shorter (*p* < 0.001 overall and in pairwise comparison) in the PSMA-I&T group (median 63 min) than in PSMA-617 (median 76 min) and PSMA-11 groups (median 71 min).

### 2.2. Distribution

**Figure 1:**
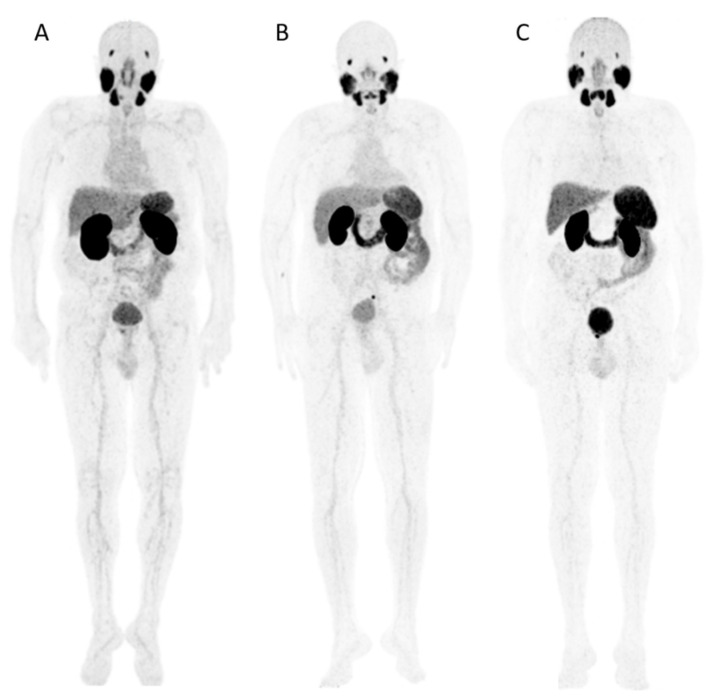
Examples of patients receiving [^68^Ga]Ga-PSMA-617 (**A**), [^68^Ga]Ga-PSMA-I&T (**B**) and [^68^Ga]Ga-PSMA-11 (**C**). The maximum intensity projections of PET imaging show intense tracer concentration in kidneys, salivary glands, spleen and duodenum as a similarity of the three radiopharmaceuticals. Every patient shows a solitary focus of pelvic tumor recurrence adjacent to the urinary bladder with individual uptake intensity. The blood pool activity of the three tracers is even visibly different, with [^68^Ga]Ga-PSMA-11 exhibiting lowest accumulation (C).

**Figure 2:**
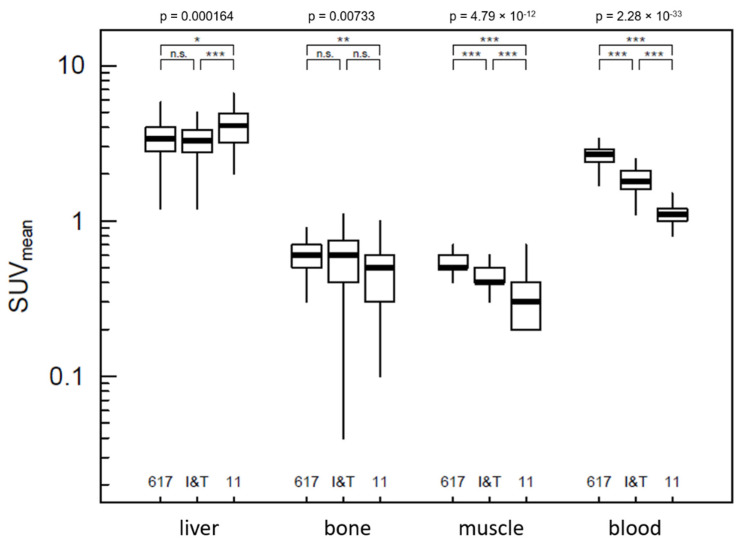
Quantitative comparison of uptake measured by SUV_mean_ within the background compartments. The *p*-values above the frame represent the comparison of the overall model between the three tracers (ANOVA); brackets demonstrate pairwise comparisons (*p* ≥ 0.05 = n.s.; *p* < 0.05 = *; *p* < 0.01 = **; *p* < 0.001 = ***).

Although there were no major differences, the biodistribution of the tracers showed minor but definite quantitative deviations (Figure 1). With regard to the background compartments with primarily unspecific uptake, the comparison revealed significant differences for the model for each of the regions examined: liver, bone, muscle and blood, *p* < 0.01 in each case (Figure 2). In contrast, the comparisons of the individual groups only revealed significant deviations in some cases. [^68^Ga]Ga-PSMA-11 showed significantly higher liver uptake than [^68^Ga]Ga-PSMA-617 and [^68^Ga]Ga-PSMA-I&T, and significantly lower bone uptake than PSMA-617. Considering musculature and blood, the uptake of all three tracers differed significantly in the same order, PSMA-617 > PSMA-I&T > PSMA-11, *p* < 0.001 in each case. All background compartments had an uptake below an SUV_mean_ of 10.

**Figure 3:**
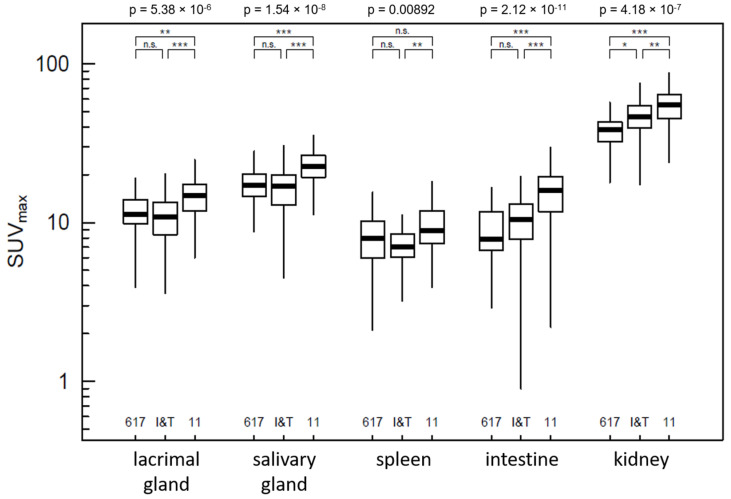
Quantitative comparison of uptake measured by SUV_max_ within the physiologically PSMA-positive organs. The *p*-values above the frame represent the comparison of the overall model between the three tracers (ANOVA); brackets demonstrate pairwise comparisons (*p* ≥ 0.05 = n.s.; *p* < 0.05 = *; *p* < 0.01 = **; *p* < 0.001 = ***).

Regarding the physiologically PSMA-positive organs (lacrimal glands, salivary glands, spleen, intestine and kidneys), the models indicateed significant differences, *p* < 0.01 in each case (Figure 3). PSMA-11 had the highest uptake in every organ except for the spleen, where uptake of PSMA-11 was not significantly higher than the uptake of PSMA-I&T. Exclusively for the renal uptake, which showed the highest SUV_max_ of all organs, PSMA-617 and PSMA-I&T differed from each other, with higher uptake of PSMA-I&T.

### 2.3. Tumor Lesions

In 132 of 190 patients, tumor lesions were detected on PET/CT. Eighteen patients (9%) had local recurrence only, 28 patients (15%) had local recurrence and metastatic spread and 86 patients (45%) had metastases only. Of the 114 patients having metastases, 90 only had one affected organ system and 24 had multiple organ systems with metastases.

**Figure 4:**
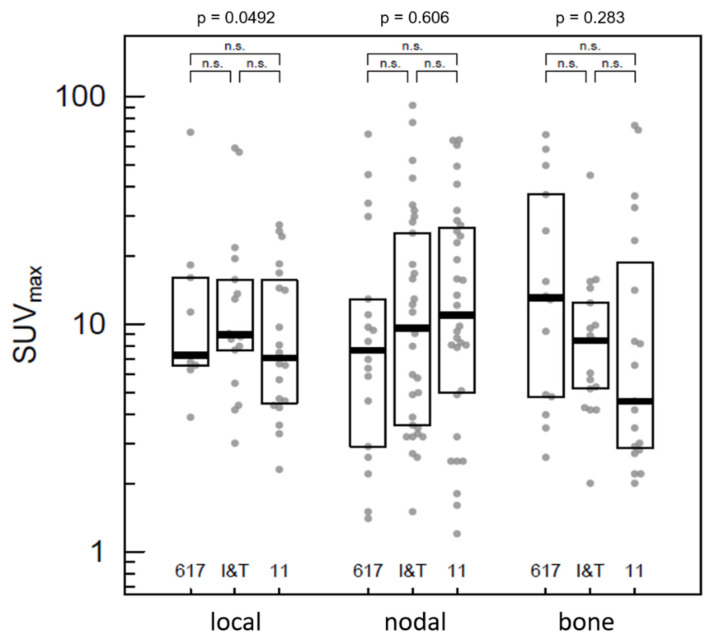
Quantitative comparison of uptake measured by SUV_max_ within the tumor lesions divided by local recurrence, nodal metastases and bone metastases. Instead of whiskers, all single values are displayed in light grey to demonstrate their variability. The *p*-values above the frame represent the comparison of the overall model between the three tracers (ANOVA); brackets demonstrate pairwise comparisons (*p* ≥ 0.05 = n.s.).

In the category-wise assessment, 186 representative tumor lesions were evaluated: 46 local lesions, 83 lymph node metastases, 50 bone metastases and 7 visceral metastases. No significant difference was found in uptake of local tumor recurrence of PSMA-617 (*n* = 9), PSMA-I&T (*n* = 17) and PSMA-11 (*n* = 20). The mean SUV_max_ was 16.2 ± 20.6 for PSMA-617, 15.7 ± 16.8 for PSMA-I&T and 10.6 ± 8.0 for PSMA-11. The same was true for the lymph node metastases, with 18, 33 and 32 lesions, respectively; and for bone metastases, with 14, 17 and 19 lesions, respectively (Figure 4). Visceral metastases were not reliably comparable due to a low number of lesions (0, 4 and 3, respectively).

### 2.4. Detection Rate

A structural correlate of PSA relapse (i.e., a local recurrence and/or metastasis) was found in 69% of patients, whereas PET/CT remained without conclusive result in 31% of cases. In the overall cohort, the probability of detecting recurrence was higher in patients after radiation (87%) than in patients after prostatectomy (66%, *p* = 0.027, OR 3.471 (1.155–10.431)), as well as in patients with prior ADT (83%) than in patients without ADT (61%, *p* = 0.002, OR 3.066 (1.514–6.208)), and in patients with Gleason score > 8 (84%) than in patients with Gleason score ≤ 7 (61%, 0.001, OR 3.293 (1.601–6.777)). In subgroups concerning the PSA level, the detection rate was significantly higher in patients with higher PSA levels. That is, the detection of recurrence was more probable in patients with PSA > 1 ng/mL (86%) than in patients having PSA ≤ 1 ng/mL (47%, *p* < 0.001, OR 7.091 (3.528 to 14.254)). This was also valid for the comparison between PSA > 0.5 ng/mL (84%) and PSA ≤ 0.5 ng/mL (38%, *p* < 0.001, OR 9.004 (4.454 to 18.203)) and the comparison between PSA > 2 ng/mL (92%) and PSA ≤ 2 ng/mL (54%, *p* < 0.001, OR 8.280 (3.303 to 20.754)).

**Figure 5:**
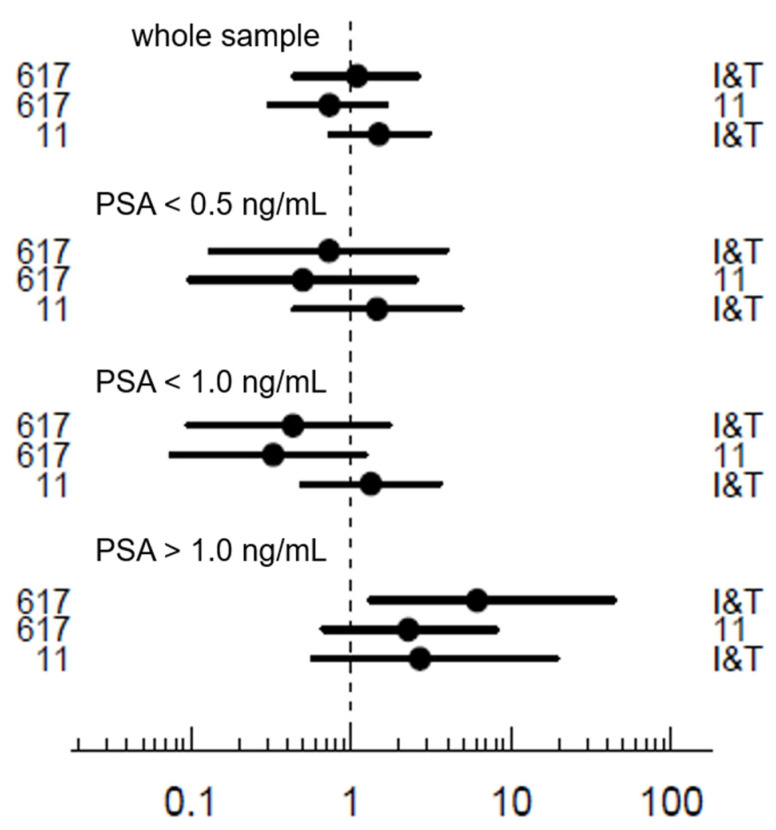
Odds ratios of detection rates as pairwise comparison of the tracers for the whole sample and relevant subgroups of PSA levels.

The detection rate was 72% in PSMA-617, 74% in PSMA-I&T and 65% in PSMA-11, without showing significant differences (*p* = 0.846, 0.461 and 0.265 in pairwise comparison, Figure 5). Regarding the Gleason score, there was no significant difference for the detection rate of the tracer groups for Gleason ≤ 7 (*p* = 0.753, 0.848 and 0.535 in pairwise comparison). However, a significantly higher detection rate was seen for PSMA-I&T in compared to PSMA-11 (*p* = 0.028) for patients with Gleason score > 8, while the other comparisons did not differ significantly. Regarding the initial treatment, no superior tracer was found for prior prostatectomy (*p* = 0.905, 0.229 and 0.187 in pairwise comparison) or for prior radiation (*p* = 0.341, 0.216 and 0.804 in pairwise comparison). Within PSA level subgroups (Figure 5), detection rates did not differ significantly between the tracers for PSA ≤ 0.5 ng/mL (*p* = 0.442, 0.328 and 0.800 in pairwise comparison), or for PSA ≤ 1 ng/mL (*p* = 0.094, 0.061, 0.799 in pairwise comparison). In contrast, PSMA-I&T showed a higher detection rate than PSMA-617 in patients with PSA > 1 ng/mL (*p* = 0.033, OR 4.071 (1.176 to 16.539)). There was no significant differences in other pairwise comparisons (*p* = 0.346 and 0.145).

## 3. Discussion

### 3.1. Limitations and Methods

This is a retrospective, inter-individual comparison, not a matched-pair study. The individual groups are heterogeneous and of limited and variable size (the PSMA-617 group in particular had few individuals). In the historical context, the selection of different tracers was based on the availability or unavailability of radiopharmaceuticals and was neither purposive nor randomized.

No strict classification of biochemical recurrence was applied; patients with PSA persistence were included in the analysis, as well as patients with high PSA levels. Balancing was sought by subgroup analyses for particularly relevant disease situations and to homogenize the groups. Differences between groups are conceivable despite similar patient characteristics, particularly due to the timing of clinical use. The presumed increase in expertise in the assessment of PET/CT images was compensated by a study-specific reappraisal of all examinations by a single experienced nuclear medicine specialist. Nevertheless, patient selection in the clinical context may have changed over time.

A gold standard for diagnostic verification (e.g., histological confirmation) of findings classified as tumor lesions by PET/CT is not available as there are local or systemic treatment options without resection of the tumor. Therefore, false-positive findings cannot be excluded [27]. Lesions that were not prostate cancer manifestations could have influenced the comparison of detection rate between the tracers. However, a systematic error is not necessarily to be expected, since to our knowledge there is no evidence for a disproportionate marking of false-positive lesions by one of the analyzed PSMA ligands. Due to biochemical tumor detection, all PET/CT examinations without a correlate are considered false-negative, demonstrating the limitations of the method in general. Irrespective of these limitations, PSMA-PET/CT is regarded as superior to conventional diagnostics and as an important factor for subsequent treatment and outcome [9,28]. Despite the scientific limitations, the practical data collected from the context of clinical routine for this medically relevant patient group and question allows direct transfer to patient care. 

In addition to the tumor-specific uptake, the physiological uptake of the tracers in organs and tissues was particularly considered and analyzed. The low tumor mass in the context of the specific study question suggests a largely physiological and unimpeded tracer distribution. Nevertheless, an impact of a tumor sink effect in patients with several tumor lesions cannot be completely excluded, although systemic bias is not necessarily expected due to similar patient characteristics of the groups, especially with regard to PSA levels. On the one hand, the relevance of these background compartments refers to the influence on the tumor-to-background ratio as a possible surrogate of detectability. On the other hand, they serve as reference parameters for the comparison between different examinations of the same patient using one or more tracers. The potential overestimation of SUV due to the chosen reconstruction method has limited relevance, as all examinations were performed on the same PET scanner with identical reconstruction parameters.

### 3.2. Results

The distribution of [^68^Ga]Ga-PSMA tracers is well studied and already known, with high concentration in kidneys and salivary glands and moderate accumulation in various other organs [11,29]. The relatively high renal binding of PSMA-11 has been reported and attributed to specific cortical binding and slower renal clearance for this compound [30].

In principle, high tumor-specific binding with low binding in tumor-free organs and physiological tissues is desirable. The diagnostically preferred ligand PSMA-11 shows higher accumulation in lacrimal and salivary glands, intestine and liver, and thus adherent or intraparenchymal metastases would potentially be more difficult to detect, which could be relevant for liver metastases in particular. However, this is not a common metastatic site of prostate cancer [31]. In contrast, the lower uptake of PSMA-11 in the skeleton compared with PSMA-617 represents a theoretical advantage for the detection of very common bone metastases. The tumor-to-background ratio compared with blood pool or soft tissue appears to be specifically relevant for the detection of local recurrence and lymph node metastases, which could potentially be superior for PSMA-11 with lower blood and muscle accumulation, but without showing impact on detection rate in the data presented here. A prior study showed concordant results regarding beneficial lesion contrast for PSMA-11 compared to PSMA-I&T, and suggested slightly better diagnostic accuracy in intra-individual comparison [24]. On the basis of the data presented here, it is not possible to determine whether any of the tracers might have detected more or additional lesions, because of inter-individual comparison. Hypothetically, the high organ binding of PSMA-11 could be disadvantageous with respect to radiation exposure and toxicity during PSMA-RLT, but for structural radiopharmaceutical reasons this ligand is not used for RLT [32,33]. For a potential comparison between follow-up studies of the same patient using different [^68^Ga]Ga-PSMA tracers, the equivalent distribution of the radiopharmaceutical can only be determined to a limited extent on the basis of the reference organs, since almost all of them differed with respect to their SUV. Only the spleen appeared to be relatively constant in its uptake between the PSMA tracers studied here and may represent a useful reference parameter.

Tracer accumulation in the detected tumor manifestations proved to be extremely heterogeneous for all ligands and manifestation sites (range of SUV values up to a factor of 100), which does not enable significant discrimination between the tracers. The SUV might have been influenced by differences in tumor lesion sizes due to partial volume effects. Furthermore, this heterogeneity prevented comparison of the tumor-to-background ratio of the tracers, since the accumulation in the background compartments deviated in a very narrow range. 

The detection rates of the analysis shown here were rather moderate, especially at low PSA levels, but high detection rates (84%) could be reliably obtained at PSA values > 0.5 ng/mL, comparable to previous studies of other authors [25,34]. Improvement of the detection rate has far-reaching consequences, as the detection of localized or solitary tumor recurrence may discriminate between curative and palliative therapeutic approaches in clinical decision making [1,9,18,35]. Therefore, the selection of the optimal ligand for PET/CT diagnostics is of interest. Since previously used choline derivatives have been widely replaced scientifically and [^18^F]F-PSMA tracers are (currently) almost exclusively available in facilities with cyclotron-based in-house manufacturing [36,37], [^68^Ga]Ga-PSMA tracers remain highly relevant and thus optimizations within this tracer group are still of high clinical importance. Implications for patient outcome cannot be made due to the inter-individual, nonrandomized comparison and heterogeneous patient characteristics of these data.

With regards to the confounders PSA level, Gleason score and initial therapy, significant differences between the groups could be excluded and thus comparability could be ensured. In addition, the relevant subgroups were compared separately to minimize the influence of the confounders. Prior ADT had a significant impact on detection rate, as it increased the probability, as previously shown in other studies [34]. The higher proportion of patients with prior ADT in the group of patients receiving PSMA-617 might have led to an overestimation of the detection rate of this tracer. The impact of the significantly shorter uptake time in the PSMA-I&T group on biodistribution and detection rate cannot be estimated with certainty, but may have led to an underestimation of this tracer with respect to detection rate, as previous studies have demonstrated an increased detection rate and uptake on later images [38,39].

Despite the differences in biodistribution, no superior tracer in terms of detection rate could be identified. In particular, no superiority of the now routinely established PSMA-11 was shown. This result is consistent with a study that found similar detection probabilities for [^68^Ga]Ga-PSMA-11 and [^68^Ga]Ga-PSMA-I&T [40]. Conversely, all three investigated ligands appear to be equally suitable for the diagnosis of biochemical recurrence, despite the current preference for PSMA-11 in routine diagnostic use. Especially in view of possible future supply shortages or production restrictions, diagnostic interchangeability without disadvantages may be of relevance. Taking special account of the subgroups characterized as diagnostically challenging with very low PSA levels, previous prostatectomy and Gleason score ≤ 7, there were no significant differences between the tracers. Only within the subgroup of patients with PSA > 1.0 ng/mL was the detection rate of PSMA-I&T significantly higher than that of PSMA-617, which should be carefully interpreted due to the small number of patients and the high statistical dispersion.

To date, no comparison of the three available [^68^Ga]Ga-PSMA ligands is available; even in meta-analyses, no comparison of the radiopharmaceuticals studied here has been published [22], emphasizing the importance of this work. For clinical application, availability rather than diagnostic validity has historically determined the selection of PET tracers. In this context, PSMA-617 does not represent a classical diagnostic tracer, but is particularly relevant for use in [^225^Ac]Ac- and [^177^Lu]Lu-PSMA RLT [41,42,43]. Using the same ligand for diagnostics and therapy has potential advantages with regard to therapy planning and pre-therapeutic dosimetry. However, this does not apply to the patient group of biochemical relapses, for whom PSMA-RLT is not the treatment of first choice.

## 4. Materials and Methods

**Figure 6:**
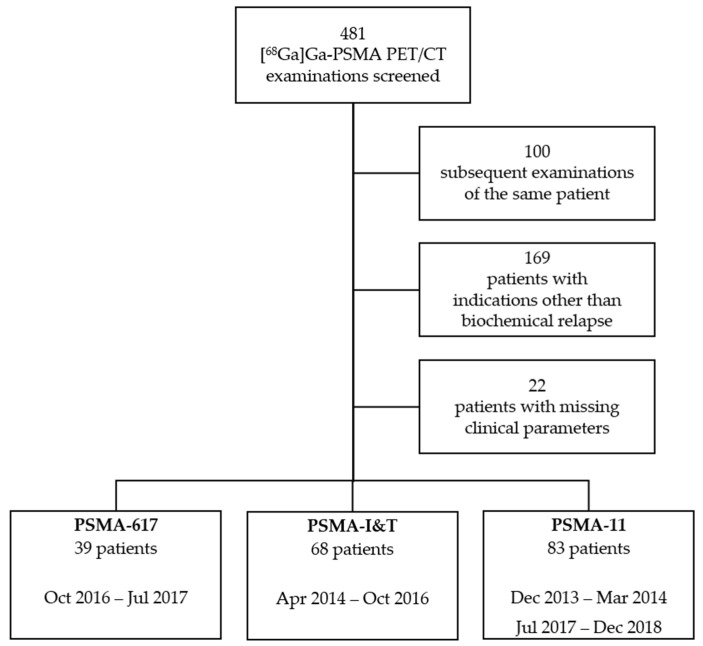
Flowchart of [^68^Ga]Ga-PSMA ligand PET/CT examinations screened and excluded based on study criteria. Patients within the three groups and associated time intervals in which the respective tracer was used.

A single-center, retrospective study is presented, including consecutive patients who underwent PSMA ligand PET/CT in a university hospital over a 5-year period (between December 2013 and December 2018). We screened 481 clinically indicated PSMA-PET/CT examinations of 381 patients (Figure 6). All patients gave full informed consent on clinical diagnostics and scientific evaluation. The institutional Ethics Committee approved the study (registration number: 2021-2209). The following inclusion criteria were applied:

- Histologically confirmed diagnosis of prostate cancer.
- Initial curatively intended therapy.
- Relapse or persistence of elevated PSA level without conclusive structural correlate in standard imaging.
- Completeness of clinical information (e.g., PSA level, Gleason score, initial and additive therapy).
- Evaluation of only the first PET/CT a patient received in the assessed interval (if multiple PET/CT were performed).

No limitations were made regarding age, initial tumor stage (except for initial metastases that would have prevented curative therapy), interval since first diagnosis, prior additional therapy (e.g., salvage radiotherapy), ongoing androgen deprivation therapy (ADT), currentness of PSA level or if patients had an ambiguous clinical suspicion of tumor lesions in prior diagnostics. Every patient was included only once, and therefore tracer comparison was inter-individual.

All three radiopharmaceuticals were produced in a GMP-certified, in-house radiopharmaceutical laboratory by radiolabeling the tracers with ^68^Ga eluted from a ^68^Ge/^68^Ga generator. Before use, all radioactive tracers were tested for radiochemical identity and purity using validated analytical methods. All injection solutions had radiochemical purities >97%. According to internal standard operating procedures, an intravenously administered activity of 250 MBq was intended (adjustments to body weight were made if necessary); additionally, 20 mg of furosemide was injected; uptake time until PET/CT scan was at least 45 min. All scans were performed at the same Biograph mCT 40 (Siemens Healthineers, Erlangen, Germany) using identical reconstruction parameters (TrueX, iterations: 3, subsets: 24, FWHM: 5 mm, matrix: 200 × 200). PET reconstruction did not meet the EARL harmonization criteria because there was no specification for ^68^Ga-radiopharmaceuticals at the time of study. The patients were scanned from feet to vertex. A secondary assessment of all examinations was carried out by a single nuclear medicine specialist with 5 years of experience in the interpretation of [^68^Ga]Ga-PSMA ligand PET/CT.

To evaluate tracer distribution the following organs and compartments (provided if present) were assessed in every patient by spherical volume-of-interest (VOI) measurements using maximum or mean standardized uptake values (SUVs) by syngo.via (Siemens Healthineers, Erlangen, Germany). SUV_mean_ was assessed by a VOI as large as anatomically possible. Typical PSMA ligand-binding organs were assessed by SUV_max_ measurements: lacrimal gland, submandibular gland representing salivary glands, spleen, duodenum representing intestine and kidney. Background compartments were covered by SUV_mean_: liver, os ilium representing bone, gluteal muscle representing musculature and cavity of ascending aorta representing blood. Tumor lesions were characterized as local recurrence (if localized in the prostatic fossa), lymph node metastasis, bone metastasis or visceral metastasis according to clinical appraisal of the observer. Only one tumor lesion was assessed per category—the one with the highest uptake. SUV_max_ of tumor lesions was ascertained. All pathologically PSMA-expressing lesions were rated as tumor lesions, if false-positive findings were not clinically obvious. 

Statistics: Analyses were conducted using the free programming language for statistical computing and graphics, R (R Core Team 2020, version 4.0.3). The normal distribution of parameters was tested prior to further analysis. Patient characteristics were compared by chi-square test for nominal variables and ANOVA for metric variables. The differences in uptake of organs, compartments and tumor lesions were examined using a univariate ANOVA; *p*-values of the overall model were quoted. A post-hoc Tukey’s HSD test was performed for pairwise comparisons. Odds ratios (ORs) with 95% confidence intervals were calculated for comparison of detection rates between tracers, concerning the whole groups and sub-groups. Pairwise comparison was reported in the order: 617 vs. I&T, 617 vs. 11, 11 vs. I&T. Level of significance was set to *p* = 0.05 for all tests.

## 5. Conclusions

PSMA ligand PET/CT with clinically widely used ^68^Ga-labeled tracers is a valuable method in detecting the structural correlate of biochemical relapse in prostate cancer. A significant difference in biodistribution between [^68^Ga]Ga-PSMA-617, [^68^Ga]Ga-PSMA-I&T and [^68^Ga]Ga-PSMA-11 was verified with potentially superior characteristics for PSMA-11 regarding blood pool persistence and background tissue accumulation. Nevertheless, none of the tracers proved to be superior in detection rate in this retrospective assessment—neither in the whole cohort nor in clinically relevant subgroups. Therefore, particularly in cases of limited availability, any of the listed PSMA ligands is feasible for PET/CT examinations in this scenario.

## Figures and Tables

**Table 1:** Patient characteristics of the three PSMA tracer groups. *p*-Values were calculated by ANOVA or chi-square test. SD, standard deviation; IQR, interquartile range (Q_3_–Q_1_).

		All (*n* = 190)	PSMA-617 (*n* = 39)	PSMA-I&T (*n* = 68)	PSMA-11 (*n* = 83)	*p*-Value
**Age** (years)	mean	69.2	69.8	68.6	69.6	0.554
	SD	6.7	7.9	6	6.6	
	median	70	71	69	70	
	range	48–82	48–81	55–82	56–81	
**Gleason** **score**	median	7	7	7	7	0.240
	IQR	1	2	1	1	
	range	4–10	4–9	5–10	4–9	
**PSA level** (ng/mL)	mean	4.5	7.1	4.5	3.3	0.214
	SD	11.2	17.8	10.7	6.7	
	median	1.4	1.6	1.3	1.3	
	range	0.01–105.3	0.05–105.3	0.01–60.3	0.03–44.2	
**Initial therapy**	prostatectomy	159 (83.7%)	32 (82.1%)	58 (85.3%)	69 (83.1%)	0.894
	radiation	31 (16.3%)	7 (17.9%)	10 (14.7%)	14 (16.9%)	
**ADT**	yes	75 (39.5%)	22 (56.4%)	24 (35.3%)	29 (34.9%)	0.053

## Data Availability

Data is contained within the article.
